# Effects of transforming growth factor-β1 on the proliferation and invasion of the HTR-8/SVneo cell line

**DOI:** 10.3892/ol.2014.2451

**Published:** 2014-08-18

**Authors:** YANZHEN ZUO, ZHIHUA FU, YATAO HU, YUHONG LI, QIAN XU, DAYONG SUN, YUSI TAN

**Affiliations:** 1 Department of Pharmacology, Chengde Medical University, Chengde, Hebei 067000, P.R. China; 2 Department of Nursing, Chengde Nursing Vocational College, Chengde, Hebei 067000, P.R. China; 3 Departments of Pathophysiology, Chengde Medical University, Chengde, Hebei 067000, P.R. China; 4 Research Laboratory, Chengde Medical University, Chengde, Hebei 067000, P.R. China; 5 Department of Tumor Radiation and Chemotherapy Center, Chengde Central Hospital, Chengde, Hebei 067000, P.R. China

**Keywords:** HTR-8/SVneo, transforming growth factor-β1, trophoblast cell, proliferation, invasion, change

## Abstract

Transforming growth factor-β1 (TGF-β1) is involved in the regulation of trophoblast cell proliferation and invasion. However, the mechanism underlying this process remains unknown, which is predominantly due to the difficulty in obtaining and maintaining primary trophoblast cells in culture over a long period of time. The HTR-8/SVneo cell line is an immortalized trophoblast cell line, which has been reported to exhibit a number of similar characteristics to those of parental trophoblast cells. Therefore, the cell line has been a useful tool for the investigation of placental function and tumor progression. In the present study, the HTR-8/SVneo cell line was used as a model to investigate the TGF-β1/SMAD signaling pathway in the proliferation and invasion of trophoblast cells. The proliferation and invasion ability of HTR-8/SVneo cells was determined using the MTT and Transwell assays, respectively. In addition, reverse transcription polymerase chain reactions were performed to detect the mRNA expression of a panel of known downstream mediators of TGF-β1, including TGF-β receptor I (TβRI), SMAD4, SMAD3, SMAD7 and tissue inhibitor of metalloproteinases-1 (TIMP-1). The results indicated that TGF-β1 promotes the proliferation and invasion of the HTR-8/SVneo cell line at passage 90. Furthermore, the expression of TβRI, SMAD3 and SMAD4 were reduced following treatment with TGF-β1, while the expression of SMAD7 was increased and the expression of TIMP-1 remained unchanged following TGF-β1 treatment. These observations indicated that the effects of TGF-β1 on the proliferation and invasion of the HTR-8/SVneo cell line at passage 90 were different from those of parental trophoblasts, which is in contrast to the results of previous studies. It was concluded that the HTR-8/SVneo cell lines, which have been grown for over 90 passages, do not accurately represent parental trophoblast cells in studies of the TGF-β/SMAD signaling pathway.

## Introduction

The proliferation and invasion of the first trimester human trophoblast cell is important in embryonic development. During the first trimester of human pregnancy, extravillous trophoblasts (EVTs) grow out from anchoring villi, invade the maternal decidua and remodel the uterine spiral arteries. Furthermore, deficient EVT invasion is associated with complications during pregnancy, including intrauterine growth restriction and pre-eclampsia (1). Abnormal proliferation and invasion of trophoblast cells may lead to gestational trophoblastic diseases, which encompass a spectrum of associated diseases, including hydatidiform mole, invasive mole, choriocarcinoma and placental-site trophoblastic tumor (2).

The proliferation and invasion of the first trimester human trophoblast cell is influenced by multiple regulatory factors, including growth factors, cytokines, adhesion molecules, proteases, matrix-derived components and oxygen tension (3,4). Transforming growth factor-β1 (TGF-β1) is involved in trophoblast proliferation and invasion (5–7). Previous studies have revealed that on the one hand, the proliferation and invasion of choriocarcinoma may be inhibited by TGF-β1 (5–12); however, on the other hand, TGF-β1 may enhance normal trophoblast functions (9,11–12). TGF-β receptor I (TβRI) and SMADs are the key downstream mediators of transcriptional responses to TGF-β. TGF-β activates TβRI and TβRII, which results in the phosphorylation of receptor-regulated SMAD2/3 proteins, which are associated with the common mediator, SMAD4. The SMAD2/3/4 complex translocates to the nucleus, binds DNA and regulates the transcription of a number of genes (13). TGF-β signaling is known to be involved in the regulation of proliferation, differentiation and apoptosis of numerous cells (14).

It is difficult to study the proliferation and invasion of human trophoblast cells *in vivo*, therefore the analysis of the growth and invasion of trophoblast cells in culture is essential for understanding the functions of the placenta. Two types of trophoblast cell models have been identified: Primary cell models and trophoblast cell lines. However, studies using primary cell culture are hindered by practical issues. For example, it is difficult to obtain the normal first trimester human chorionic tissues, and the number and purity of primary trophoblasts available are limited. Furthermore, cultures become contaminated with other placental cell types, including fibroblasts (15). In addition, trophoblast primary cells cannot be maintained for long time periods in culture. Therefore, these cells are not suitable for studies involving genetic manipulations that often require long-term cell culture (12). The HTR-8/SVneo cell line was established by introducing the gene encoding simian virus 40 large T antigen into first trimester human trophoblasts. It is an immortalized trophoblast cell line, which exhibits a number of similar characteristics to those of parental trophoblast cells (12). Graham *et al* (12) revealed that HTR-8/SVneo cells were inhibited by recombinant TGF-β1, which is identical to that of the parental trophoblast cells (12). Therefore, in the present study, the HTR-8/SVneo cell line was selected to investigate the TGF-β/SMAD signaling pathway and the involvement of such in the proliferation and invasion of trophoblast cells.

The proliferation of HTR-8/SVneo cells was investigated using MTT assays and the invasion ability was determined by Transwell assay, following the incubation of cells with various concentrations of TGF-β1. In addition, the mRNA expression levels of TβRI, SMAD4, SMAD3, SMAD7 and tissue inhibitor of metalloproteinases-1 (TIMP-1) were examined to elucidate which factor leads to the abnormal regulation exhibited by TGF-β1. The implications of the results and comparison with previous data have been discussed.

## Materials and methods

### Cell culture

HTR-8/SVneo cells (the 90th passage) were provided by Queen’s University at Kingston (Kingston, Canada). The cells were cultured in an incubator with an atmosphere of 5% CO_2_ at 37°C in RPMI-1640 medium (Hyclone, Waltham, MA, USA) supplemented with 10% fetal bovine serum (FBS; Hangzhou Sijiqing Biological Engineering Materials Co, Ltd, Hangzhou, China), 1 mM pyruvic acid sodium salt, 2 mM glutathione, 100 U/ml penicillin and 100 μg/ml streptomycin. The cells were then subcultured with 0.25% trypsin and 0.02% EDTA (Sigma-Aldrich, St. Louis, MO, USA) when the cell growth reached 70–80%, and the density of subcultured cells was 1:3.

### Analysis of cell viability by MTT assay

A total of 1×10^5^ cells/ml in 200-μl aliquots were plated in 96-well plates and allowed to adhere overnight. Next, the cells were incubated for 24, 48 and 72 h with or without various concentrations of TGF-β1 (200 μl for a final concentration of 0, 0.05, 0.5, 5, 10, 12.5, 25, 50, 100 and 200 μg/l; six wells for each concentration; PeproTech Inc., Rocky Hill, NJ, USA). The cell viability was determined using MTT reagent (Gibco-BRL, Carlsbad, CA, USA) and the absorbance was determined at a wavelength of 492 nm using a microplate reader (Multiskan MK3; Thermo Fisher Scientific, Waltham, MA, USA). The experiment was repeated five times.

### Transwell invasion assay

A thin layer of growth factor-reduced diluted Matrigel (BD Biosciences, Franklin Lakes, NJ, USA) was added to the upper chambers of 6.5-mm Transwell inserts with polycarbonate membrane filters containing 8-μm pores (Corning Inc, Acton, MA, USA). Inserts were placed into 24-well culture plates and incubated at 37°C for 4 h. Next, 500 μl aliquots of RPMI-1640 supplemented with 20% FBS were added to the lower chambers. Concurrently, 1×10^5^ cells/ml in 200 μl aliquots of serum-free RPMI-1640 containing 0, 1, 10 and 100 μg/l TGF-β1, respectively, were added to the upper chambers of the inserts and cultured for 48 h. Cells remaining on the upper surface of the Matrigel layer were removed using a cotton swab and dried, and the cells that had invaded the bottom of the membrane were fixed in 4% paraformaldehyde for 10 min and stained with hematoxylin. The invasive cells were observed under a light microscope (Nikon 80i; Nikon, Tokyo, Japan) at ×100 magnification. Images of five random fields were captured for each membrane and the average number of cell numbers detected in each field was calculated. The total number of transmigrated cells was counted by selecting 10 random fields and observing the number of apoptotic cells using a light microscope (magnification, ×200; BH-2; Olympus Corporation, Tokyo, Japan).

### Reverse transcription-polymerase chain reaction (RT-PCR)

A total of 3×10^5^ cells were plated in six-well plates and incubated for 24 h, followed by culturing the cells with serum-free RPMI-1640 for 24 h. Following cell synchronization, the medium was changed and added to TGF-β1 with 0, 1, 10 and 100 μg/l, respectively, as the final concentrations to incubate the cells for an additional 48 h. Total RNA from each group of cells was isolated using TRIzol reagent (Invitrogen Life Technologies, Carlsbad, CA, USA) according to the manufacturer’s instructions. Next, 1% agarose gel electrophoresis was performed to separate the RNA and 28S, 18S and 5S bands were detected. The M-MLV first-strand synthesis system kit (Invitrogen Life Technologies) and 1 μg RNA with oligo(dt)20 primers were used to synthesize the cDNA. The Taq kit [Takara Biotechnology (Dalian) Co., Ltd., Dalian, China] was used for PCR amplification. The primers used are shown in Table I. β-actin was used as the internal control (16). The amplification of cDNA was performed over varying cycles: 94°C for 2 min, 30 cycles at 94°C for 30 sec, followed by the indicated annealing temperature (Table I) for 30 sec, and 72°C for 1 min. The PCR products were electrophoresed in 2% agarose gel, images were captured using the ZF ultraviolet transmission reflection analyzer (Shanghai Jiapeng Technology Co., Ltd., Shanghai, China) and gray values were measured using Quantity One-4.6.2 software (Bio-Rad, Hercules, CA, USA). The relative level of the target mRNA expression was defined as the ratio of the absorbance of the target band to the β-actin band.

### Statistical analysis

Statistical analysis was performed using SPSS software, version 19.0 (IBM Corporation, Armonk, NY, USA). All data are expressed as the mean ± standard deviation. Multifactorial analysis was used to analyze the results of the MTT assay, and one-way analysis of variance was used for other comparisons. The pairwise comparisons of several means between groups was performed using the Student-Newman-Keuls method. P<0.05 was considered to indicate a statistically significant difference.

## Results

### Effects of TGF-β1 on the proliferation of HTR-8/SVneo cells

**Figure 1:**
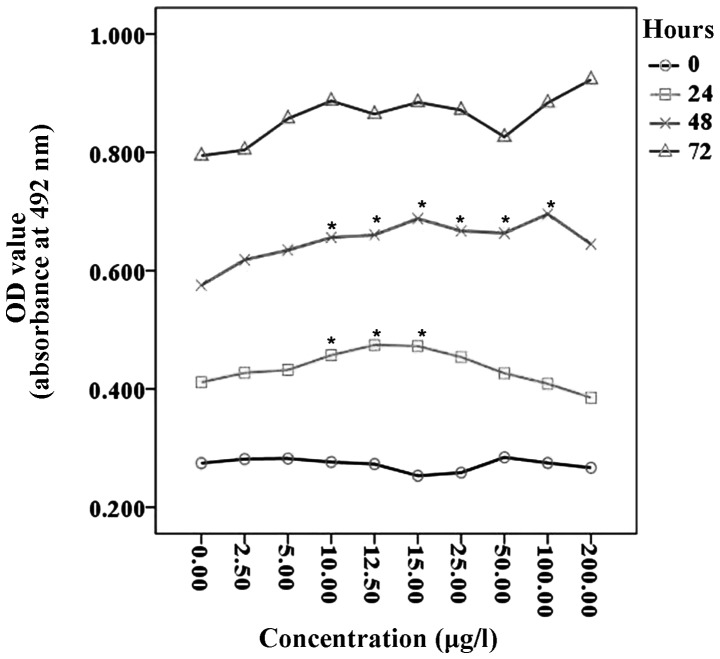
Effects of TGF-β1 on the proliferation of HTR-8/SVneo cells. Cell viability was measured by MTT assay (n=6) and the experiment was repeated five times. Multifactorial analysis was used, and no proliferation was detected when the HTR-8/SVneo cells were incubated for 0 h with or without various concentrations of TGF-β1. Following incubation with TGF-β1 for 24 h, a significant increase of HTR-8/SVneo cells when compared with control cells was identified, in particular for cells grown with 10–15 μg/l TGF-β1 (P<0.05). Following incubation with TGF-β1 for 48 h the proliferation was significantly increased with 10–100 μg/l TGF-β1 (P<0.05). In addition, an improved proliferation was also observed in cells incubated with TGF-β1 for 72 h; however, no significant difference was identified when compared with the 0 μg/l group (^*^P<0.05). OD, optical density; TGF-β1, transforming growth factor-β1.

An MTT assay of the HTR-8/SVneo cells was performed to examine cell proliferation in the presence of various concentrations of recombinant TGF-β1. No proliferation was identified when the HTR-8/SVneo cells were incubated for 0 h with or without various concentrations of TGF-β1. Following incubation with TGF-β1 for 24 h, a significant increase in HTR-8/SVneo cells was identified, when compared with control cells, in particular for cells grown with 10–15 μg/l TGF-β1 (P<0.05). Following incubation with TGF-β1 for 48 h, the proliferation of HTR-8/SVneo cells was significantly increased with 10–100 μg/l of TGF-β1 (P<0.05). In addition, an increased level of proliferation was observed in cells incubated with TGF-β1 for 72 h; however, the increase was not statistically significant (P>0.05; Fig. 1).

### Effects of TGF-β1 on the invasion of HTR-8/SVneo cells

**Figure 2:**
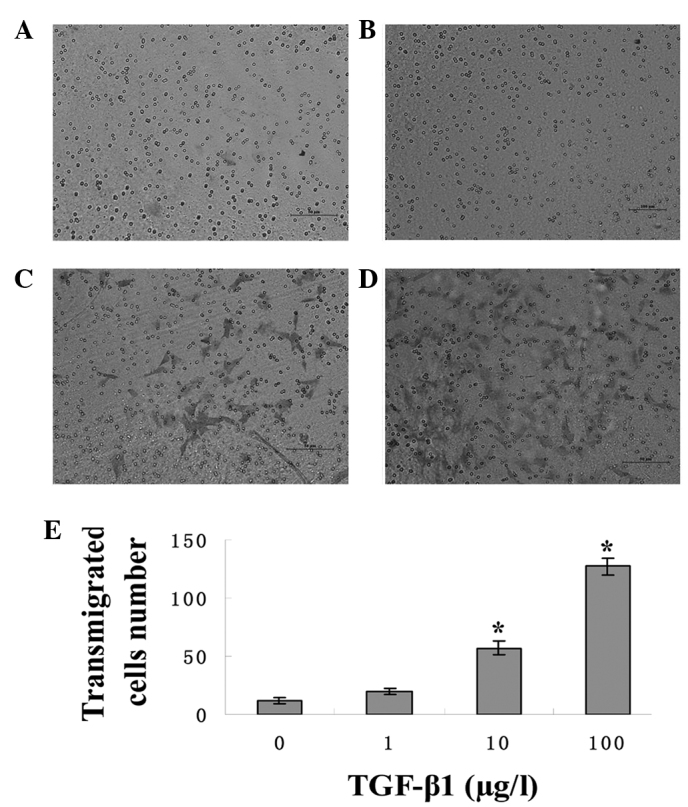
Effects of (A) 0, (B) 1, (C) 10 and (D) 100 μg/l TGF-β1 on the invasion of HTR-8/SVneo cells (magnification, ×100). The invasion ability was compared by treating the cells with 0, 10 or 100 μg/l of TGF-β1 for 48 h. (E) The results indicated that the cells grown in the presence of 10 and 100 μg/l TGF-β1 transmigrated more than the cells grown in the absence of TGF-β1, indicating an increased invasion (P<0.05). The most efficacious concentration of TGF-β1 with regards to increased cell invasion was 100 μg/l (P<0.05). Data are presented the mean ± SD (n=5). ^*^P<0.05 vs. control group. TGF-β1, transforming growth factor-β1.

The effect of TGF-β1 on the invasion ability of the HTR-8/SVneo Cells was examined using Transwell invasion assays. The invasion ability of the cells was compared by treating the cells with 0, 10 or 100 μg/l of TGF-β1 for 48 h. The results demonstrated that cells grown in the presence of 10 and 100 μg/l TGF-β1 transmigrated more than the cells grown in the absence of TGF-β1, which indicated an increased invasion (P<0.05). The most efficacious concentration of TGF-β1 with regard to increased cell invasion was 100 μg/l (P<0.05) (Fig. 2).

### Regulation of TβR1, SMAD4, SMAD3, SMAD7 and TIMP-1 mRNA expression by TGF-β1 in HTR-8/SVneo cells

**Figure 3:**
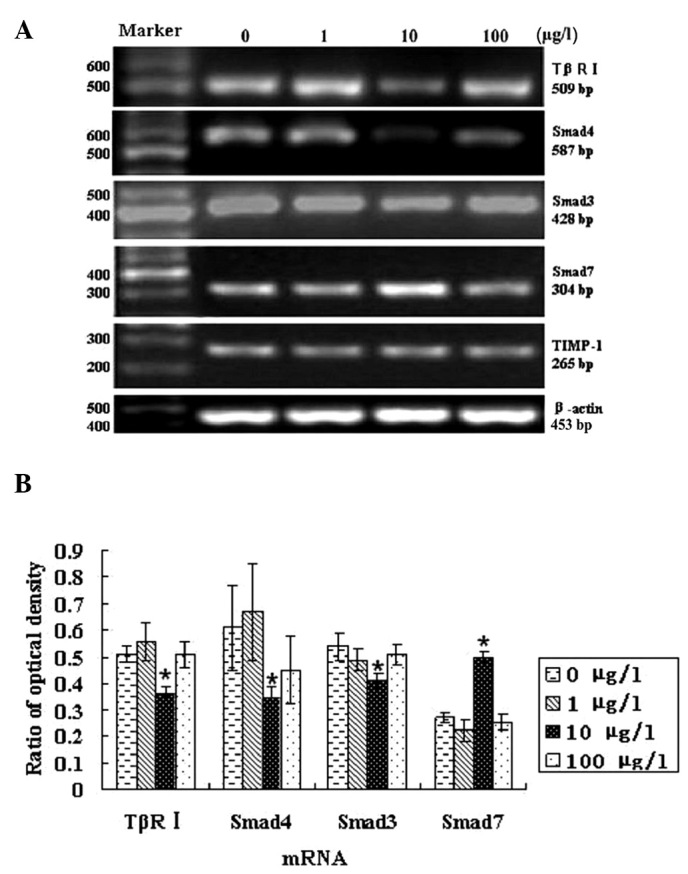
Regulatory effects of varying doses of TGF β1 for 48 h on TβR1, SMAD4, SMAD3, SMAD7 and TIMP-1 mRNA in HTR 8/SVneo cells. (A) TβR1, SMAD4, SMAD3, SMAD7 and TIMP-1 mRNA expression by TGF-β1 in HTR 8/SVneo cells. (B) RT-PCR analysis of TβR1, SMAD4, SMAD3, SMAD7 and TIMP-1 mRNA levels by TGF-β1 in HTR 8/SVneo cells (mean ± SD; n=5). The expression of TβRI, SMAD4 and SMAD3 mRNA was decreased, whereas that of SMAD7 mRNA increased in cells grown with 10 μg/l of TGF-β1 (P<0.05). However, no significant differences were identified between the mRNA expression in the four genes in cells grown in the presence of 1 or 100 μg/l TGF-β1 (P>0.05). In addition, the expression of TIMP-1 mRNA remained unchanged in the presence of all TGF-β1 doses applied in this study (P>0.05). ^*^P<0.05, vs. the control group. TβR1, transforming growth factor β receptor I; TIMP-1, tissue inhibitor of metalloproteinases-1; TGF-β1, transforming growth factor-β1.

Subsequently, the mRNA expression of the known TGF-β1 downstream mediators was compared using RT-PCR in the HTR-8/SVneo cells by treating these cells with various TGF-β1 concentrations for 48 h. The results indicated that the expression of TβRI, SMAD4 and SMAD3 mRNA was decreased and that of SMAD7 mRNA was increased in cells grown in the presence of 10 μg/l of TGF-β1 (P<0.05). However, no significant difference in the mRNA expression of the four genes was identified in cells grown in the presence of 1 or 100 μg/l TGF-β1 (P>0.05). In addition, no significant differences were identified in the expression of TIMP-1 mRNA in the presence of any of the doses of TGF-β1 applied (P>0.05; Fig. 3).

## Discussion

It is generally accepted that the proliferation and invasion of the first trimester human trophoblast cells is important in embryonic development. Primary cell and transformed trophoblast cell models have been developed to investigate the proliferation and invasion of the placenta and placental tumors, respectively. In particular, the HTR-8/SVneo cell line has been established to investigate the biology of normal trophoblast cells as they have been reported to share certain characteristics with their parental cells.

TGF-β is a family of cytokines, which are multifunctional peptides that regulate proliferation, differentiation, adhesion, migration and other functions of numerous cell types. TGF-β comprises of three isoforms: TGF-β1, β2 and β3. The normal trophoblast proliferation and invasiveness of the uterus are strictly regulated processes that are inhibited by TGF-β1 produced locally (6–9). TGF-β1 may inhibit the growth of epithelial cells and induce apoptosis, thus acting as a tumor suppressor (17). However, the TGF-β1 gene is also frequently upregulated in tumor cells, and mutations in this gene may result in Camurati-Engelmann disease (18). TGF-β1 is overexpressed in various types of malignancies. For example, Lv *et al* (19) demonstrated that TGF-β1 induced the epithelial-to-mesenchymal transition of breast cancer cells and promoted breast cancer cell metastasis (19). TGF-β1 has also been found to be overexpressed in invasive types of hepatocellular carcinoma and may be involved in the rapid progression of hepatocellular carcinoma (20). Dave *et al* (21) found that higher TGF-β1 levels were exhibited in the serum of breast cancer patients. Furthermore, Dehaghani *et al* (22) revealed that the TGF-β1 serum levels were significantly higher in gestational trophoblastic disease patients when compared with those in pregnant and non-pregnant controls.

In the current study, the proliferation of the HTR-8/SVneo cells was found to increase following incubation with TGF-β1 (10–100 μg/l) for 24 or 48 h. An increased invasion was also observed when the cells were treated with 10 or 100 μg/l TGF-β1 in the Transwell assays. Notably, previous studies have reported that TGF-β1 may significantly inhibit the cell invasion (11). In addition, Graham *et al* (12) demonstrated that HTR-8/SVneo cells were inhibited by recombinant TGF-β1, which is the same as that of parental trophoblast cells (12). Considering that the HTR-8/SVneo cell line used were over 90 passages in cell culture, the results of the present study indicated that the proliferation and invasion ability of the HTR-8/SVneo cell line has been changed over 90 passages. This observation is similar to the results of Khoo *et al* (23), which demonstrated that the immortalized trophoblast RSVT-2 and RSVT2/C cell lines were hyper-proliferative and -invasive when compared with their parental HTR8 cell line. The RSVT-2 and RSVT2/C cell lines were also resistant to the anti-proliferative and -invasive effects of TGF-β1 to different extents. Khoo *et al* (23) also revealed that the downregulation of connexins, and the resultant impairment in gap junctional intercellular communication, caused cells to escape from the inhibition of TGF-β1, which may be an early event in tumor progression, as observed in the premalignant SV40 Tag transformants (24). We hypothesized that the changes in cytokines may cause the HTR-8/SVneo cell line to escape from the inhibition of TGF-β1, and to exhibit a hyper-proliferative and -invasive phenotype following numerous passages, as observed in the current study. Therefore, further studies investigating the expression of the downstream mediators of TGF-β1 were performed.

TGF-β1 is normally inactive in cells and exerts its biological effects depending on its downstream molecules. Any change occurring to its downstream mediators leads to the dysregulation of TGF-β1, which is illustrated by the following examples. The expression levels of TGF-β1 and TGF-βR-1 genes have been demonstrated to be higher in the gastric cancer tissues (25). Exogenous glucosamine promotes the osteogenic differentiation of human dental pulp stem cells via increasing the levels of TGF-βRI and phosphorylated SMAD2 (26). The expression of SMAD3, SMAD4 and phosphorylated SMAD3, as well as TGF-βR type I and type II, were all higher in leiomyoma when compared with those in myometrium (27).

SMADs are important intracellular proteins, which transfer the information of TGF-β to the nucleus. In mammals, four types of SMADs have been identified: i) receptor-regulated SMADs (R-SMADs) comprising SMAD2 and SMAD3, which transduce TGF-β signaling; ii) SMAD1, SMAD5 and SMAD8, which transduce the bone morphogenetic protein signaling; iii) a common SMAD called co-SMAD4, which is a key mesomerism that associates with R-SMADs and translocates to the nucleus; and iv) inhibitory SMADs, SMAD6 and SMAD7, which compete with R-SMADs for receptor binding, thereby inhibiting the R-SMAD phosphorylation (28,29). Previous studies have demonstrated that the decreased expression levels of TβRI, SMAD3 and SMAD4 may cause cancer cells to escape the growth inhibition of TGF-β1, and the increased expression of SMAD7 may block the inhibitory effects of TGF-β1. Thus TβRI, SMAD3, SMAD4 and SMAD7 are closely associated with the development of tumors. For example, the expression of SMAD4 was lower, and that of SMAD7 was significantly higher, in the gastric cancer tissues than in the peri-tumoral tissues (30). Similarly, the expression of SMAD4 was lower, whereas SMAD7 was found to positively correlate with tumor grading in human glioma and gastric cancer (31,32). SMAD3 may mediate *in vivo* signaling that is inhibitory to epithelial wound healing and thus, during the physiological process of wound healing, the suppression of SMAD3 levels may engage (33). In the physiological tissue repair as well as the pathological fibrosis, SMAD3 mRNA was markedly downregulated and the antagonistic SMAD7 was rapidly and transiently induced by TGF-β1 to stimulate collagen synthesis (34). These results indicate that SMAD3 is involved in the growth inhibition by TGF-β. The aberrant expression of SMAD7 has been shown to be involved in inflammatory bowel disease and scleroderma (35). In addition, SMAD7 mRNA levels are increased in human pancreatic cancer. Furthermore, SMAD7 transfected colo-357 cells exhibit enhanced anchorage-independent growth and accelerated growth in nude mice (36).

The results of the present study, obtained from RT-PCR assays, revealed that when compared with that of the control cells, the mRNA levels of TβRI, SMAD3 and SMAD4 were significantly decreased in the HTR-8/SVneo cells treated with 10 μg/l TGFβ1 (P<0.05), while the mRNA level of SMAD7 was significantly increased. In addition, no evident changes in the expression of TβRI, SMAD3 and SMAD4 mRNAs were identified in cells treated with 1 and 100 μg/l TGFβ1 (P>0.05). These results indicated that the decreased mRNA expression of TβRI, SMAD3 and SMAD4 may reduce the inhibitory effect of TGF-β1 on cell proliferation, while the enhanced mRNA expression of SMAD7 further blocks the inhibitory effect of TGF-β1 on cell proliferation. This may explain the significant increase in proliferation identified when cells were treated with TGF-β1 at this concentration.

The changes in SMAD7 expression observed in the HTR-8/SVneo cell line over several passages were similar to those in the immortalized bronchial BEP2D epithelial cells. A previous study on the BEP2D cells and the radiation-induced malignant transformation of bronchial BERP35T2 epithelial cells revealed that the SMAD7 gene was highly expressed, and the cells that exhibited a high expression of SMAD7 demonstrated an active proliferative capacity. In addition, the inhibitory effect of TGF-β1 on the cell growth of these cells was weak. Furthermore, the changes in SMAD7 expression were associated with the malignant transformation of bronchial epithelial cells and the development of lung cancer (37).

In the present study, the mRNA expression of the TIMP-1 gene that encodes a natural inhibitor of the matrix metalloproteinases (MMPs) was also examined by RT-PCR. MMPs are a family of >23 zinc-binding enzymes, which are involved in the proteolytic degradation of the extracellular matrix. MMP-9 and -2 have been shown able to mediate the invasion of trophoblast cells, and the natural tissue inhibitors of MMP, including the TIMP family, inhibit their invasiveness (38). The results of the present study indicated that the mRNA levels of TIMP-1 were not altered. However, the mechanism underlying the increased invasion of the HTR-8/SVneo cells regulated by TGF-β1 requires further investigation.

In conclusion, the cell lines may be convenient for studies; however, the cell lines expressed a number of novel characteristics (39–42). The results of this study indicate that the effects of TGF-β1 on the proliferation and invasion of the HTR-8/SVneo cell line at passage 90 were different from those of the parental trophoblasts, which is in contrast to the results of previous studies. We hypothesized that HTR-8/SVneo cell lines, which have been grown for over 90 passages do not accurately represent parental trophoblast cells for studies of the TGF-β/SMAD signaling pathway.

## Figures and Tables

**Table I:** Primers used in the polymerase chain reaction.

Target	Primer sequences (5′-3′)	Product size (bp)	Annealing temperature (°C)	Cycles, n
TβRI	F:GGC CAA ATA TCC CAA ACA GAT R: AAT CCA ACT CCT TTG CCC TTA	509	60	35
Smad3	F: ACA GCT GTG TCT GCC AAA CAC R: ATG TTC TGA GAG GGG AGG GAG	428	59	30
Smad4	F: CAG CAT CCA CCA AGT AAT CGT R: CTC TCA ATG GCT TCT GTC CTG	587	60	30
Smad7	F:CCT CCT TAC TCC AGA TAC CCA R:ACC AGC TGA CTC TTG TTG TCC GAA T	304	55	30
TIMP-1	F:GTT GTT GCT GTG GCT GAT AG R:TGT GGG ACC TGT GGA AGT A	265	58	30
β-actin	F: AGC GGG AAA TCG TGC GTG AC R: ACA TCT GCT GGA AGG TGG AC	453	58	30

TβRI, transforming growth factor β receptor I; TIMP-1, tissue inhibitor of metalloproteinases-1; F, forward; R, reverse.
